# Supplementary material for: Genome assembly and association tests identify interacting loci associated with vigor, precocity, and sex in interspecific pistachio rootstocks
Source: G3 (Bethesda). 2022 Dec 1;13(2):jkac317. doi: 10.1093/g3journal/jkac317 (PMC9911073; doi:10.1093/g3journal/jkac317)
Supplement: jkac317_Supplementary_Data [file jkac317_supplementary_data.zip › Supplemental_Table_Legends_G3-2022-403626.docx]

**Supplementary Table Legends**

**Table S1.** Details of external data used in this study.

**Table S2.** Estimates of genome size and heterozygosity from genomescope and flowcytometry.

**Table S3.** Full contig and scaffold statistics for each stage of the assembly process, and BUSCO gene set completeness statistics for each assembly and transcriptome.

**Tables S4**. Marey maps for *P. integerrima* and *P. atlantica*.

**Table S5**. Complete phenotyping data set as used in this study.

**Tables S6**. SNPs significantly associated with sex, as detected using the *P. integerrima* and *P. atlantica* reference assemblies.

**Tables S7.** SNPs significantly associated with various phenotypes in the experimental orchard dataset, as detected using the *P. integerrima* and *P. atlantica* reference assemblies.

**Tables S8.** SNPs significantly associated with rootstock diameter in the commercial orchard dataset, as detected using the *P. integerrima* and *P. atlantica* reference assemblies.

**Table S9.** Reference and alternate nucleotides at significant markers.

**Table S10.** ANOVA results for the interaction effect of rootstock diameter associated SNPs identified on Chromosomes 3 and 9, and η^2^ and R^2^ estimates for proportion of variance in rootstock diameter explained by SNPs identified on Chromosomes 3 and 9.

**Tables S11.** List of candidate genes and putative SNP effects for *P. integerrima* Chromosome 3 and *P. atlantica* Chromosome 9.

**Table S12.** Chi-square contingency table and p values for genotypic ratios, by orchard, at Chromosome 3 and Chromosome 9
