# Supplementary figures and images for: Genome assembly and association tests identify interacting loci associated with vigor, precocity, and sex in interspecific pistachio rootstocks
Source: G3 (Bethesda). 2022 Dec 1;13(2):jkac317. doi: 10.1093/g3journal/jkac317 (PMC9911073; doi:10.1093/g3journal/jkac317)

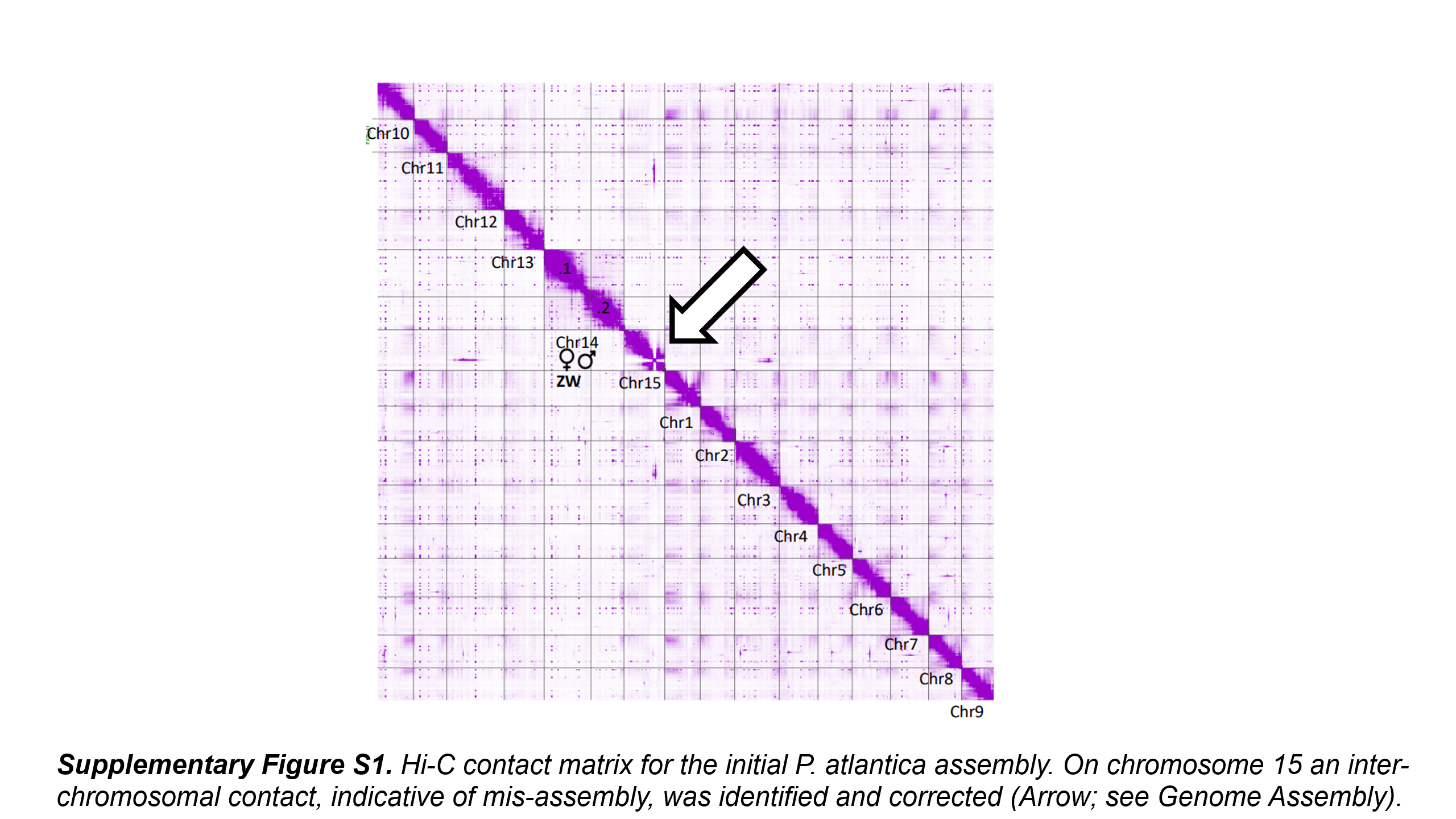

Supplement: jkac317_Supplementary_Data [file jkac317_supplementary_data.zip › Supplementary_Figure_S1_G3-2022-403626.tif]

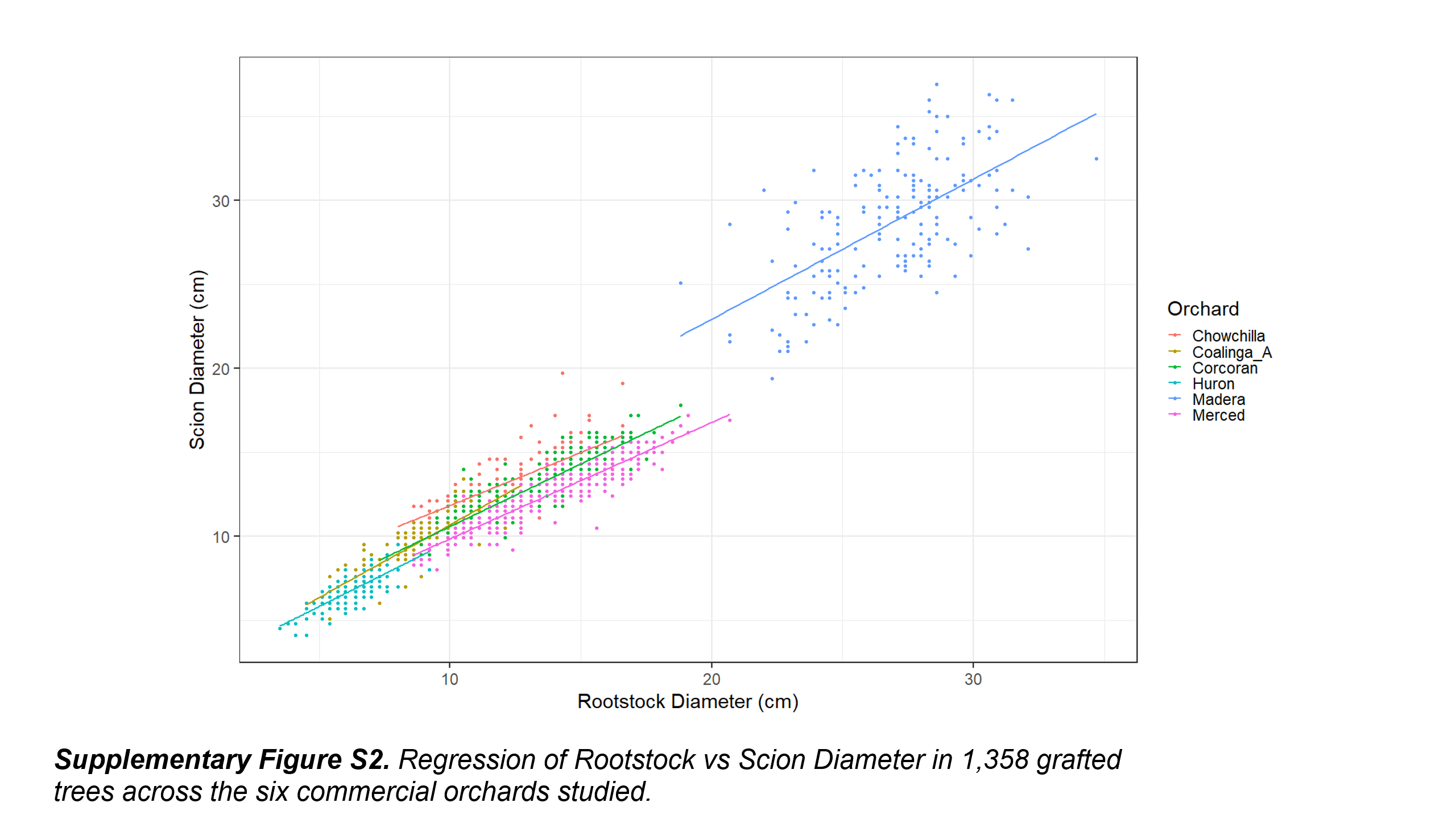

Supplement: jkac317_Supplementary_Data [file jkac317_supplementary_data.zip › Supplementary_Figure_S2_G3-2022-403626.tif]

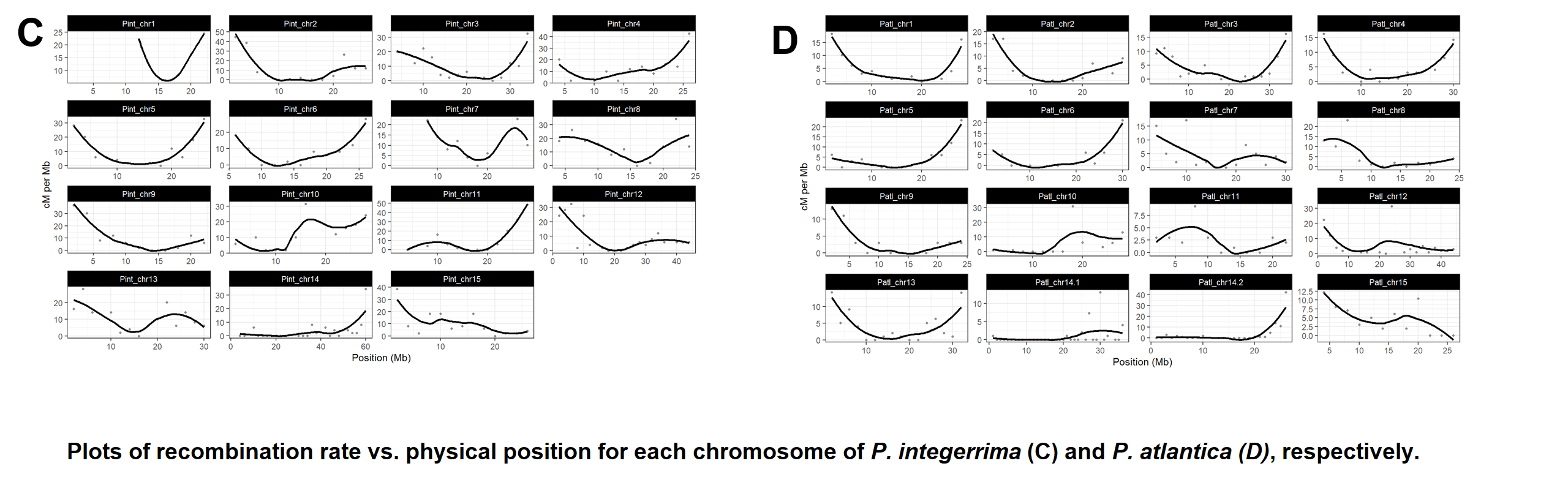

Supplement: jkac317_Supplementary_Data [file jkac317_supplementary_data.zip › Supplementary_Figure_S3_G3-2022-403626.tif]

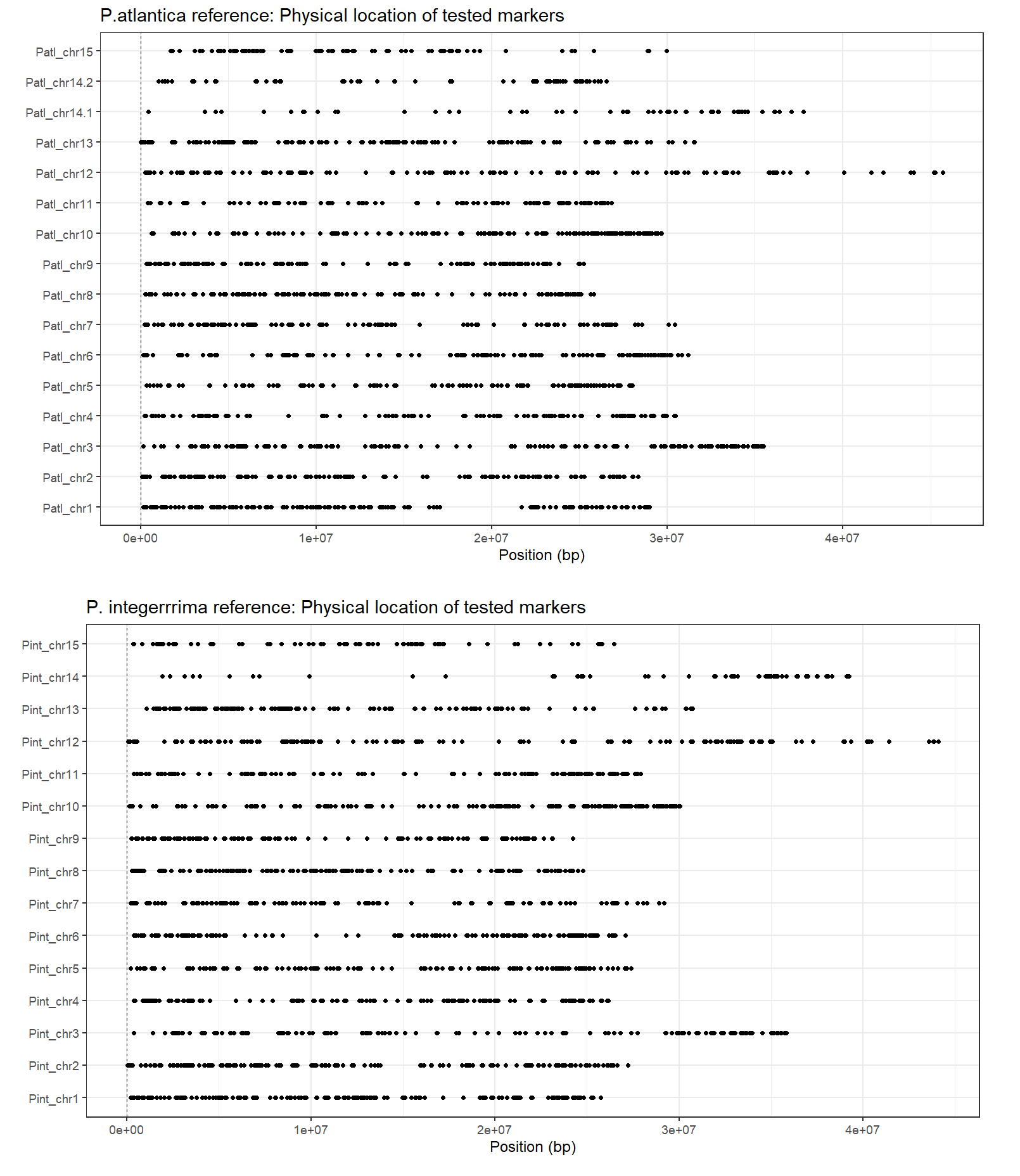

Supplement: jkac317_Supplementary_Data [file jkac317_supplementary_data.zip › Supplementary_Figure_S4_G3-2022-403626.tif]
